# The Parkinson's Disease Genome‐Wide Association Study Locus Browser

**DOI:** 10.1002/mds.28197

**Published:** 2020-08-31

**Authors:** Francis P. Grenn, Jonggeol J. Kim, Mary B. Makarious, Hirotaka Iwaki, Anastasia Illarionova, Kajsa Brolin, Jillian H. Kluss, Artur F. Schumacher‐Schuh, Hampton Leonard, Faraz Faghri, Kimberley Billingsley, Lynne Krohn, Ashley Hall, Monica Diez‐Fairen, Maria Teresa Periñán, Jia Nee Foo, Cynthia Sandor, Caleb Webber, Brian K. Fiske, J. Raphael Gibbs, Mike A. Nalls, Andrew B. Singleton, Sara Bandres‐Ciga, Xylena Reed, Cornelis Blauwendraat, the International Parkinson's Disease Genomics Consortium (IPDGC)

**Affiliations:** ^1^ Laboratory of Neurogenetics National Institute on Aging, National Institutes of Health Bethesda Maryland USA; ^2^ Data Tecnica International Glen Echo Maryland USA; ^3^ German Center for Neurodegenerative Diseases Tubingen Germany; ^4^ Lund University Translational Neurogenetics Unit, Department of Experimental Medical Science Lund Sweden; ^5^ Universidade Federal do Rio Grande do Sul Hospital de Clínicas de Porto Alegre Porto Alegre Brazil; ^6^ Department of Human Genetics McGill University Montreal Quebec Canada; ^7^ Department of Molecular and Clinical Pharmacology Institute of Translational Medicine, University of Liverpool Liverpool UK; ^8^ Fundació Docència i Recerca Mútua Terrassa and Movement Disorders Unit, Department of Neurology University Hospital Mútua Terrassa Barcelona Spain; ^9^ Unidad de Trastornos del Movimiento, Servicio de Neurología y Neurofisiología Clínica, Instituto de Biomedicina de Sevilla Hospital Universitario Virgen del Rocío/CSIC/Universidad de Sevilla Seville Spain; ^10^ Lee Kong Chian School of Medicine Nanyang Technological University Singapore Singapore Singapore; ^11^ Human Genetics Genome Institute of Singapore, A*STAR Singapore Singapore; ^12^ UK Dementia Research Institute, Cardiff University Cardiff UK; ^13^ The Michael J. Fox Foundation for Parkinson's Research, Grand Central Station New York NY USA

**Keywords:** GWAS, Parkinson's disease, prioritization

## Abstract

**Background:**

Parkinson's disease (PD) is a neurodegenerative disease with an often complex component identifiable by genome‐wide association studies. The most recent large‐scale PD genome‐wide association studies have identified more than 90 independent risk variants for PD risk and progression across more than 80 genomic regions. One major challenge in current genomics is the identification of the causal gene(s) and variant(s) at each genome‐wide association study locus. The objective of the current study was to create a tool that would display data for relevant PD risk loci and provide guidance with the prioritization of causal genes and potential mechanisms at each locus.

**Methods:**

We included all significant genome‐wide signals from multiple recent PD genome‐wide association studies including themost recent PD risk genome‐wide association study, age‐at‐onset genome‐wide association study, progression genome‐wide association study, and Asian population PD risk genome‐wide association study. We gathered data for all genes 1 Mb up and downstream of each variant to allow users to assess which gene(s) are most associated with the variant of interest based on a set of self‐ranked criteria. Multiple databases were queried for each gene to collect additional causal data.

**Results:**

We created a PD genome‐wide association study browser tool (https://pdgenetics.shinyapps.io/GWASBrowser/) to assist the PD research community with the prioritization of genes for follow‐up functional studies to identify potential therapeutic targets.

**Conclusions:**

Our PD genome‐wide association study browser tool provides users with a useful method of identifying potential causal genes at all known PD risk loci from large‐scale PD genome‐wide association studies. We plan to update this tool with new relevant data as sample sizes increase and new PD risk loci are discovered. © 2020 The Authors. *Movement Disorders* published by Wiley Periodicals LLC on behalf of International Parkinson and Movement Disorder Society. This article has been contributed to by US Government employees and their work is in the public domain in the USA.

Parkinson's disease (PD) is a multifactorial disease for which both genetic and environmental risk factors play a role. In the past decade, approximately 20 genes have been associated with PD or parkinsonism in families.^1^ More than 90 common variants have been associated with sporadic PD risk, age at onset, and progression using genome‐wide association studies (GWASes).^2^, ^3^, ^4^, ^5^

One major challenge remaining after GWAS identification of risk loci is the localization and characterization of specific causal variant(s) and gene(s) at each locus. A common misconception is that the most significant GWAS variant exerts an effect on the nearest gene (as commonly reported in GWAS articles), but this is unlikely to be the case. First, the GWAS variant is not necessarily causative on its own, but is instead likely to tag a functional region or variant in high linkage disequilibrium (LD). Second, variants in noncoding regions containing regulatory sequences may impact distant genes by altering 3‐dimensional chromatin conformation, placing these genes outside the predefined LD region.^6^, ^7^ Several approaches can be taken when prioritizing genes for each significant variant. For PD, these approaches include using single‐cell RNA‐seq to determine gene expression in relevant cell populations,^8^ transcriptome‐wide association studies,^9^ and quantitative trait loci (QTL).^3^ Others have functionally prioritized a single locus (*SNCA* ^10^ and *TMEM175* ^11^), but these functional single‐locus experiments often do not scale up to loci with many genes. Other non‐disease‐specific pipelines have been developed using epigenetic and chromatin conformation data sets in addition to expression QTL data^12^, ^13^; however, some disease‐specific interpretation is required. Therefore, we have aggregated multiple data sets from several sources to create a versatile and user‐friendly tool (https://pdgenetics.shinyapps.io/GWASBrowser/) to prioritize specific genes and variants for additional PD GWAS and functional studies that aim to identify potential therapeutic targets (Fig. 1).

**FIG. 1:**
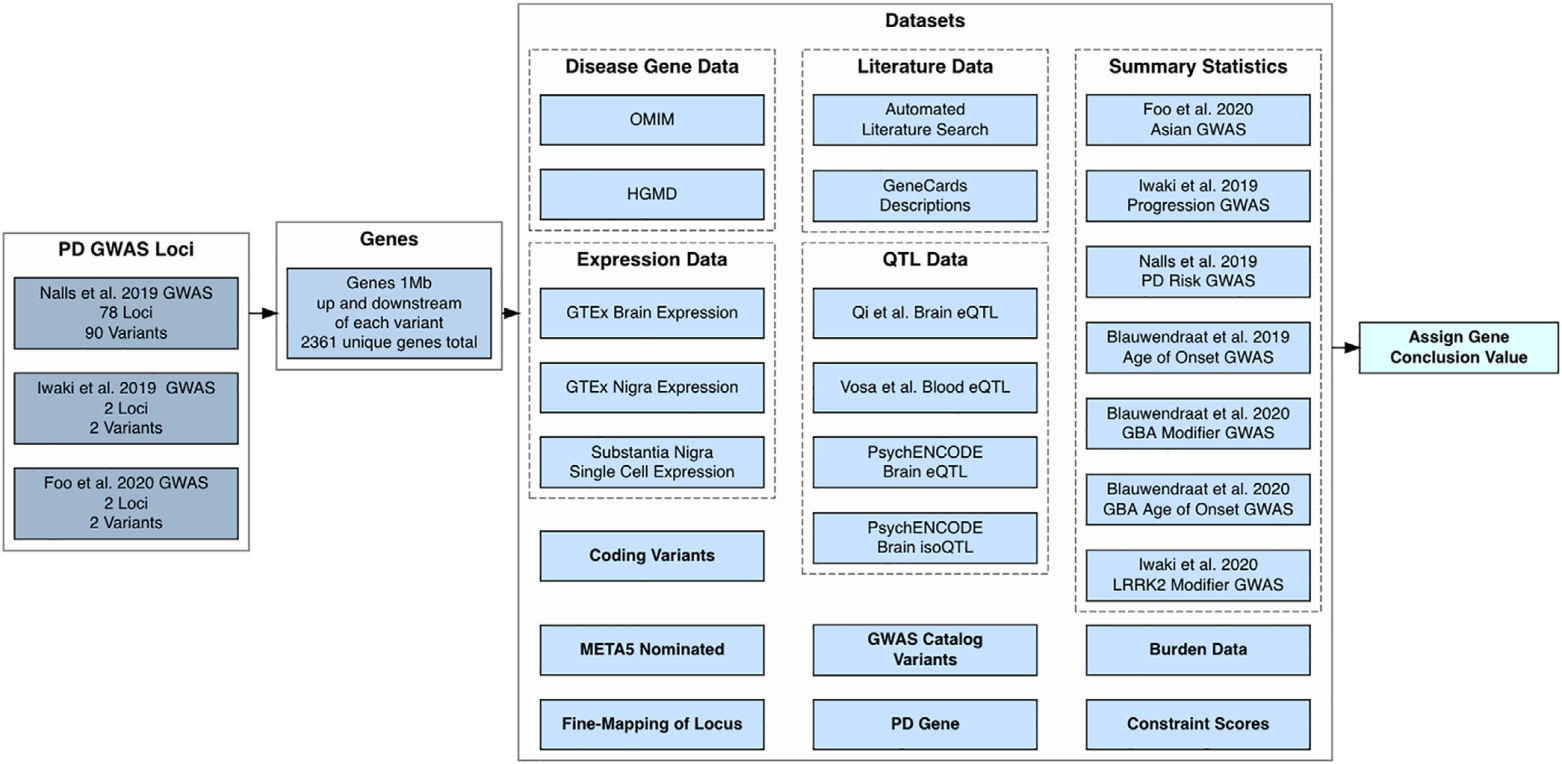
Flowchart of data gathered for the browser. Summary of the variants, genes, and data sets included in the browser to prioritize genes for each locus. Data sets include genome‐wide association study (GWAS) summary statistics, known coding variants (Nalls et al, 2019),^3^ nominated genes, online Mendelian inheritance in man (OMIM), and human gene mutation database (HGMD) disease genes, expression quantitative trait locus data (eQTL), variants from the GWAS catalogue, known Parkinson's disease and related disorder genes, PubMed literature data, Genotype‐Tissue Expression (GTEx), and single‐cell expression data, burden test data, fine‐mapping data, and constraint data.

## Methods

### GWAS Loci and Gene Selection

All GWAS loci and summary statistics were gathered from our recent PD GWAS studies.^2^, ^3^, ^4^, ^5^, ^14^, ^15^ We selected all genes 1 megabase (Mb) up and downstream of each significant variant from the hg19 reference genome.^16^ Genes in this 2‐Mb range were included in locus zoom plots created for each variant.^17^

### Gene Expression Data

The Genotype‐Tissue Expression (GTEx) portal was accessed on February 12, 2020, to obtain v8 gene expression data. Transcript per million (TPM) data was averaged across all available brain and substantia nigra (SN) samples individually for each gene. Average single‐cell RNA sequencing expression data were also included for SN astrocytes, SN dopaminergic neurons, SN endothelial cells, SN GABAergic cells, SN microglial cells, SN oligodendrocyte cells, and SN oligodendrocyte progenitor cells.^18^ An arbitrary value of 5 TPM was chosen as the cutoff for significance in brain tissue, SN tissue, and SN dopaminergic neuron averages. Genes with greater than 5 TPM in any of these 3 data sets were given a value of 1 in the evidence table in the Brain Expression, Nigra Expression, or SN‐Dop. Neuron Expression columns. Genes with no available expression data were set to NA in these columns.

### Expression Quantitative Trait Loci Data

Expression quantitative trait locus (eQTL) data were collected from the summary‐data‐based Mendelian randomization (SMR) website for brain tissues^19^ and processed using SMR software tools.^20^ Blood tissue eQTL data were collected from the eQTLGen consortium.^21^ Additional brain tissue data were collected from the PsychENCODE project.^22^ Locus compare plots were generated using GWAS data and the appropriate eQTL data to compare the distribution of eQTL and GWAS data.^23^ Plots were omitted if they contained neither the locus risk variant nor a good proxy variant. Proxy variants were obtained using the LDlinkR library^24^, ^25^ in R (https://www.r-project.org/) to find variants with *r* ^2^ > 0.7 with the risk variant. Genes were given a value of 1 in the “QTL‐brain” column in the evidence table if there were sufficient data to create brain locus compare plots using Qi et al eQTL data, PsychENCODE eQTL data, or PsychENCODE isoQTL data. Genes were given a value of 1 in the “QTL‐blood” column in the evidence table if there were sufficient data in the blood tissue eQTL data to create a locus compare plot. Pearson correlation coefficients between GWAS and blood or brain eQTL/isoQTL *P* values were calculated using R for each gene with sufficient data. The “QTL‐correl” column in the evidence table is given a value of 1 if the magnitude of the correlation coefficient is greater than the user assigned cutoff (default cutoff of 0.3) in any of the gene's locus compare plots. This column was given a value of “NA” if the gene has no plots with a “QTL‐brain” or “QTL‐blood” score of 1.

### Literature Search

Automated literature searches were performed in PubMed using the search term “GENE_NAME[Title/Abstract] AND Parkinson's[Title/Abstract]” and “GENE_NAME[Title/Abstract]” to search for the respective gene and its occurrence in PD literature using the rentrez package in R.^26^ The number of search results was collected for all the genes in each locus to create a bar plot of PubMed hits for each locus. Genes with 5 or more search results in the PD and gene name search were given a score of 1 in the “Literature Search” column in the evidence table. A word cloud was generated for each gene using the PubMedWordcloud package in R.^27^ Last, gene descriptions were obtained from GeneCards.^28^

### Constraint Data

Constraint data were downloaded from the gnomAD browser (https://gnomad.broadinstitute.org/downloads).^29^ These data were included to predict how resistant genes are to variation. Constraint *z* scores were included for synonymous variants and missense variants. A probability of being loss‐of‐function intolerant (pLI) score was included for loss‐of‐function variants. Observed/expected variant values were included for these 3 variant types, along with the 90% confidence interval for each value. Genes were given a score of 1 in the “Variant Intolerant” column in the evidence table if the upper limit of the 90% confidence interval was less than 0.35 for any variant type.

### Burden Data

Burden summary statistics were obtained from the most recent GWAS^3^ and an exome sequencing study in PD (article in process). In total, 40 different burden tests on exome sequencing data and 2 different burden tests on imputed GWAS data were performed using only missense and loss‐of‐function variants with minor allele frequency cutoffs of 0.05 and 0.01. The minimum *P* values of all 40 exome burden tests were included for each gene in the burden table of the browser. The minimum *P* value for the 2 burden tests on imputed data for each gene was included in the same table. These 2 minimum *P* values were Bonferroni‐corrected by the number of genes with data in the burden test to determine significance (1480 genes for exome and 1026 genes for imputed). Genes with significant burden results in either exome or imputed data were given a value of 1 in the “Burden” column in the evidence table. Genes with no available burden data were given a value of “NA” in this column.

### Nalls et al 2019 Nominated Genes and Known Parkinson's Disease Genes

The PD risk GWAS^3^ used 4 QTL data sets to determine causal genes for each GWAS signal in the study. These data were obtained from Supplementary Table 1 of the Nalls et al 2019 GWAS paper. Seventy of the 90 variants from this study were found to be associated with a putative causal gene. These genes were given a value of 1 in the “Nominated by META5” column of the evidence table. Genes known to be monogenic for PD, parkinsonism, or other related movement disorders were given a value of 1 in the “PD Gene” column in the evidence table.^1^, ^30^

### Other Disease Genes

Disease gene data were gathered from the Human Gene Mutation Database (HGMD)^31^ and Online Mendelian Inheritance in Man (OMIM).^32^ For HGMD, only genes with variants classified as “DM” (disease‐causing mutations) were included. These genes were given a value of 1 in the “Disease Gene” column in the evidence table.

### Coding Variants

Coding variants in linkage disequilibrium (LD) with risk variants were obtained from internal databases. *R* ^2^ and D′ LD scores were calculated using PLINK.^33^ Combined annotation‐dependent depletion (CADD) scores were obtained from the CADD database using ANNOVAR.^34^ Frequencies were obtained from the gnomAD database also using ANNOVAR.^29^, ^34^

### Associated Variant Phenotypes

Phenotypes of variants in LD with risk variants were obtained from the GWAS catalog v1.0.2.^35^ *R* ^2^ and D′ LD scores were calculated using PLINK (v1.9)^36^ from a large PD case‐control reference set including over 40,000 individuals. Frequencies were obtained from the gnomAD database using ANNOVAR.^29^, ^34^

### Fine‐Mapping

Variants in the PD risk meta‐analysis summary statistics^3^ were reannotated to GRCh38p7 build positions using dbSNP build 151. If a variant's dbSNP rsid was not present in dbSNP build 151, it was excluded from further analysis. The summary statistics were partitioned into risk regions or loci based on physical distance. Per chromosome, these partitions were generated iteratively by finding the variant with the smallest *P* value and extracting this variant and those variants within 1 Mb of it. The region of extracted proximal variants was checked against other extracted regions, and if their edges were within 100 kb, the regions were merged. These iterations continued until no variant with a GWAS *P* < 5 × 10^−8^ remained within the PD meta‐analysis summary statistics.

The FINEMAP tool^3^, ^37^ was used to fine‐map these PD‐risk locus regions. Finemap uses Shotgun Stochastic Search^38^ and Bayesian Model Averaging^39^ to identify casual configurations of risk variants. The maximum number of causal variants within the configuration per locus was based on the number of independent risk signals detectable per locus. This number of independent risk signals per locus was estimated using the stepwise model selection procedure implemented in GCTA‐COJO. Both the Finemap and GCTA‐COJO^40^, ^41^ tools require linkage disequilibrium (LD) information for their search and modeling. For this purpose, TOPMed freeze5b samples of European ancestry, available from dbGaP, were used as an LD reference panel. This panel included 16,257 samples.

### Browser Design

The GWAS locus browser is an R shiny application. Data were precompiled and loaded onto the application's server and is not obtained from external databases in real time. The GWAS locus browser is an open‐source project. The code is available on our github (https://github.com/neurogenetics/GWAS_locus_browser).

## Results

### Data Browser

The PD GWAS locus browser (https://pdgenetics.shinyapps.io/GWASBrowser/) is an online platform designed to assist researchers with the prioritization of genes located within PD GWAS loci. It includes multiple layers of data, including: GWAS statistic, eQTL, burden, expression, constraint, and literature data and a flexible scoring system that users may configure for their own needs. Interestingly, several PD GWAS hits show high correlation and overlap, *R* ^2^ > 0.8 and D′ > 0.9 (Supplementary Table 1), with other disease risk signals including diseases such as inflammatory bowel disease (locus 2),^42^, ^43^ neuroticism (loci 29, 62, 69, and 73),^44^, ^45^, ^46^ body mass index (loci 4, 11, 61),^47^, ^48^ and insomnia (locus 73).^49^ Below we describe 2 use case scenarios on how this application could be used for prioritizing genes from PD GWAS loci.

### Use Case Scenario 1, Locus 16, rs11707416

**FIG. 2:**
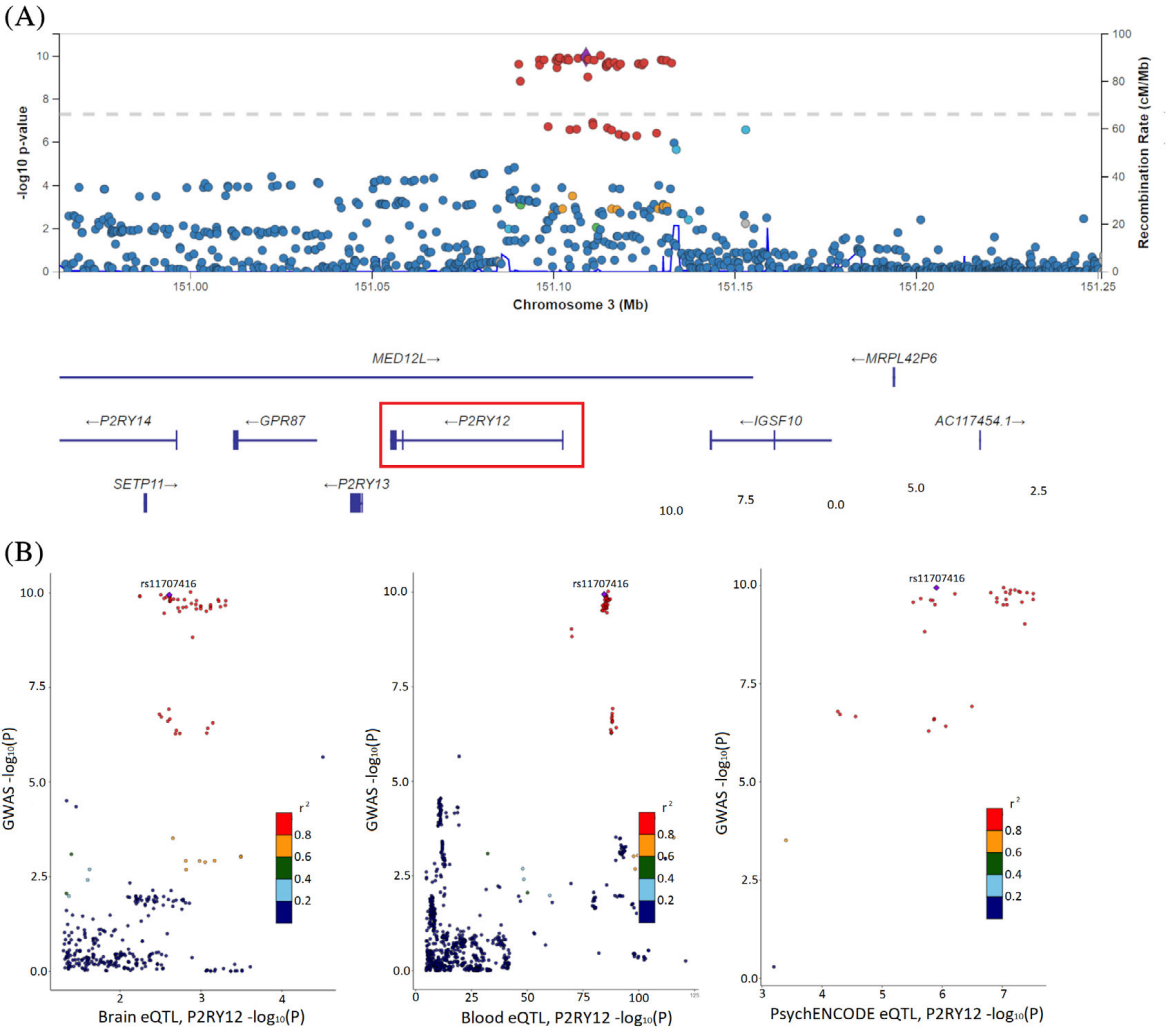
Locus zoom and locus compare plots for locus 16 and variant rs11707416 and *P2RY12*. (**A**) Locus zoom Manhattan plot for risk variant rs11707416 on locus 16. The risk variant is uniquely colored purple, and all other variants are colored by their *r* ^2^ value. Recombination rate peaks are plotted in blue. Nearby genes are included at the bottom, with *P2RY12* highlighted. (**B**) Locus compare plots for *P2RY12* on locus 16 plot –log_10_ *P* values for Nalls et al (2019) GWAS data (*y* axis) and for different eQTL data sets (*x* axis). Data sets available for *P2RY12* are Qi et al brain eQTL, Vosa et al blood eQTL, and PsychENCODE brain eQTL (left to right). Variants are colored by their *r* ^2^ value, and the risk variant is labeled and uniquely colored purple.

A possible use case for the browser exists on locus 16 for the risk variant rs11707416. This variant was discovered in the most recent PD risk GWAS (OR, 0.94; SE, 0.0097; *P* = 1.13 × 10^−10^),^3^ and the closest gene is *MED12L*. The locus zoom plot showed a clear GWAS signal for this region (Fig. 2A); however, no genes in this locus were prioritized using current methods in the PD risk GWAS. Using the default settings, *P2RY12* (purinergic receptor P2Y) has the highest conclusion score (7) in the evidence per gene table, meaning it has the highest sum of data set scores for that locus. Locus compare eQTL plots showed some correlation in both brain and blood, indicating some overlap in the distribution of eQTL and GWAS data for this gene (Fig. 2B). There is one common coding variant at this locus, but it is located within *MED12L* (mediator complex subunit 12‐like; NM_053002:exon25:c.G3629A:p.R1210Q) and not *P2RY12*. *MED12L* has a lower conclusion score (3) than *P2RY12*, suggesting that even with this coding variant, *MED12L* is not the primary candidate in this locus. This is a very complex and unusual locus, in that there are multiple genes encoded within an intron of an isoform of *MED12L* (*P2RY12, P2RY13, P2RY14, GPR171*), and even more interesting is that the missense variant that changes an amino acid of *MED12L* is located in an intron of the much smaller gene, *P2RY12* (Fig. 2A). Despite the uniqueness of the locus structure, we will focus on *P2RY12* as the primary candidate nominated by the PD GWAS browser data sets.

**FIG. 3:**
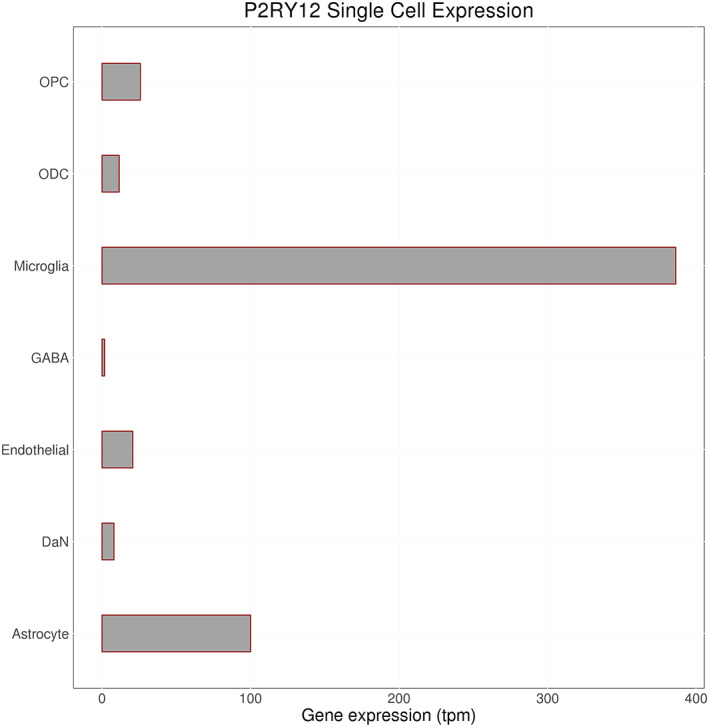
Bar plot for single‐cell expression of *P2RY12* on locus 16. Transcript per million (TPM) data for *P2RY12* was averaged across all samples for 7 cell types from the substantia nigra. These include oligodendrocyte progenitor cells (OPCs), oligodendrocyte cells (ODCs), microglia, GABAergic neurons (GABA), endothelial cells, dopaminergic neurons (DaNs), and astrocytes.

Expression of *P2RY12* was significant in all included databases (GTEx brain, GTEx SN, and single‐cell dopaminergic neuron data). However, it appears that *P2RY12* has much higher expression in astrocytes and microglia than neurons (Fig. 3). The disease gene section shows that *P2RY12* has been linked to platelet type 8 bleeding disorder.^50^ This disorder appears to be caused by dominant‐negative mutations in the *P2RY12* gene that disrupt the homodimerization of the receptor that is required for normal function.^51^ No direct links between *P2RY12* and PD have been reported in the literature, but *P2RY12* is a widely studied gene with roles suggested in neuroinflammation, apoptosis, and autophagy, pathways that are relevant to PD and other neurodegenerative diseases.^52^, ^53^, ^54^ Experiments have been done to characterize expression patterns of *P2RY12* in microglia and its role in neuroinflammation.^55^ As these experiments focused on Alzheimer's disease, it would be useful to build on them in the PD context in the future.

However, this is not conclusive evidence, and other potential candidates exist on this locus. For example, *SLENOT* has a low conclusion score (2), partly because of lack of data for the gene, but is suggested to play a protective role in PD in the literature.^56^ Overall, the possible role of *P2RY12* in PD should be further analyzed with functional experiments.

### Use Case Scenario 2: Locus 78, rs2248244

**FIG. 4:**
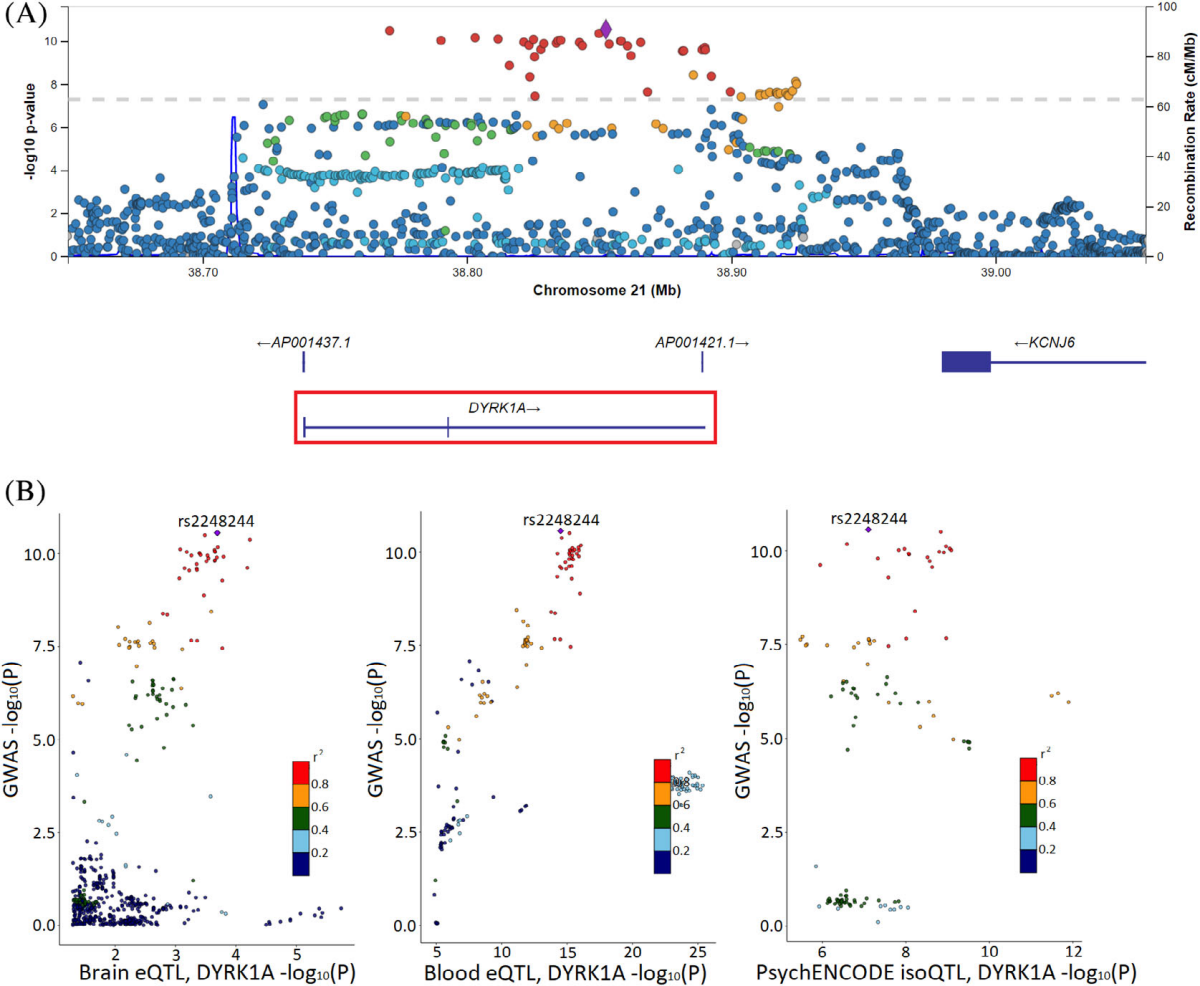
Locus zoom and locus compare plots for locus 78 and variant rs2248244 and *DYRK1A*. (**A**) Locus zoom Manhattan plot for risk variant rs2248244 at locus 78. The risk variant is uniquely colored purple, and all other variants are colored by their *r* ^2^ value. Recombination rate peaks are plotted in blue. Nearby genes are included at the bottom, with *DYRK1A* highlighted. (**B**) Locus compare plots for *DYRK1A* on locus 78 plot –log_10_ *P* values for Nalls et al (2019) GWAS data (*y* axis) and for different eQTL data sets (*x* axis). Data sets available for *DYRK1A* are Qi et al brain eQTL, Vosa et al blood eQTL, and PsychENCODE brain isoQTL (left to right). Variants are colored by their *r* ^2^ value, and the risk variant is labeled and uniquely colored purple.

We chose a second use case for the browser at locus 78 for the risk variant rs2248244. This variant was discovered in the most recent PD risk GWAS (OR, 1.074; SE, 0.0107; *P* = 2.74 × 10^−11^),^3^ and its nearest gene, *DYRK1A*, was nominated as the causal gene in that study. The locus zoom plot showed a clear GWAS signal for this region (Fig. 4A). The default settings in the evidence per gene table nominated *DYRK1A* as the top candidate with a conclusion score of 10, which is higher than any of other genes within the locus (second highest is 4).

**FIG. 5:**
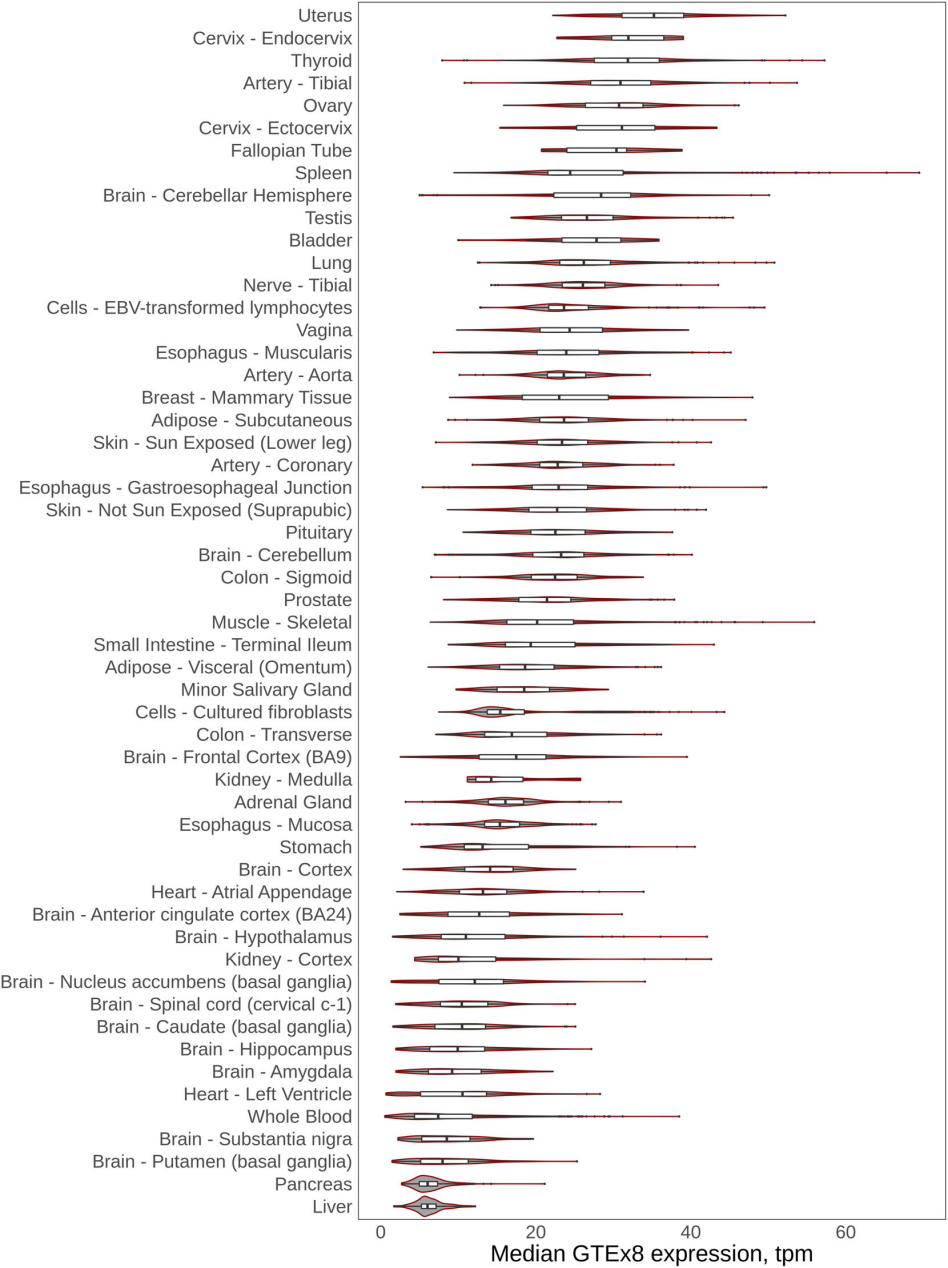
GTEx violin plot for *DYRK1A* on locus 78. *DYRK1A* transcript per million (TPM) data were averaged across all available samples from GTEx v8 data for different tissues. Distribution and probability of expression level are included for each tissue.

The brain and blood eQTL plots and the isoQTL plot showed good correlation between GWAS and QTL values (Fig. 4B). No coding variants or other known associated disease variants exist for this locus. *DYRK1A* showed significant gene expression in all databases (GTEx brain, GTEx SN, and single‐cell dopaminergic neuron data; Fig. 5). Constraint data for *DYRK1A* showed a low 90% CI for loss‐of‐function variation and a pLI of 1, suggesting significant intolerance to loss‐of‐function variation. However, burden test results showed no significant change in variants for *DYRK1A* after Bonferroni correction. FINEMAP results of this locus nominated several variants with rs2248244 and rs11701722, both intronic with the highest probability score.

*DYRK1A* is located in the Down syndrome critical region on chromosome 21, and various deletions and single‐nucleotide variants have been linked to autosomal‐dominant mental retardation‐7 (MRD7).^57^, ^58^, ^59^ These data are not enough to conclude that *DYRK1A* is the relevant risk gene at this locus. However, *DYRK1A* also has significant representation in the PD literature. Previous studies have suggested that *DYRK1A* encodes a kinase that can phosphorylate α‐synuclein and *Pink1* in mammalian cells.^60^, ^61^, ^62^, ^63^ Studies in mouse models have indicated that haploinsufficiency of *DYRK1A* leads to a reduction in dopaminergic neurons while increasing the dosage results in more dopaminergic neurons by altering apoptosis.^60^, ^61^, ^62^ All these studies combined indicate that loss of *DYRK1A* expression may influence the number of dopaminergic neurons and thereby the development of PD.

Again, this is not conclusive, and other potential candidates exist at this locus. For example, *TTC3* has significant expression data and is suggested to play a role in Alzheimer's disease^64^ and Down syndrome^65^ in the literature. Overall, the PD GWAS browser predicts *DYRK1A* is a good candidate for more functional PD experiments and high‐powered human genetic studies aimed at characterizing the molecular mechanism underlying risk at this locus.

## Discussion

GWASes have identified numerous risk loci for a large number of diseases (https://www.ebi.ac.uk/gwas/).^35^ The current bottleneck after completing these studies is identifying the causal genes and variants underlying the GWAS signals. There is a large discrepancy between the number of new GWAS loci identified and the number of studies that molecularly characterize these loci to identify a causative variant or gene. Currently, the number of PD loci that are functionally validated is very low and mostly includes genes that are known to cause monogenic forms of PD. PD GWASes have identified a number of these pleiotropic genes at various loci such as *SNCA* (locus 23), *GBA* (locus 1), *LRRK2* (locus 49), and *VPS13C* (locus 59). Current evidence suggests that *TMEM175* (locus 19^11^), *CTSB* (locus 37^9^, ^14^) and *GCH1* (locus 56^66^) are also pleiotropic and lead to PD by multiple mechanisms.

Our goal is to provide the PD research community with a tool that catalogs all significant PD GWAS signals and helps to prioritize the genes at each locus for functional studies. The overall significance score for each gene is displayed in the “Conclusion” column of the evidence table. This score is the sum of all other numerical values from each data set for the respective gene. The inclusion of genes 1 Mb up and downstream of each variant is an arbitrary cutoff and may limit the ability of the browser to accurately prioritize genes. This limitation will come into effect when variants affect genes outside this range; however, prior studies have found that functional noncoding variants are often located within this 2 Mb window. In addition, some loci contain more than 1 significant variant (eg, locus 1, *GBA* with 3 reported independent signals), and until a way to detect precise causative variants is developed, it is assumed that all significant variants within the same locus will impact the same gene.

Although all the data sets included in the PD browser may contribute to the prioritization of PD genes, each data set comes with its own limitations that should be taken into account when considering the conclusion value. Blood and brain eQTL data were included to identify genes with similar GWAS and eQTL data distribution. Although similar distributions may suggest causality, the power of these data sets may reduce their importance. Blood eQTL data will have more statistical power than brain data because of their larger sample size, but may be less relevant to PD. Another important note is that we just tested for cis eQTLs because we simply did not have the power to detect robust trans‐QTLs. Scoring for the “QTL‐brain” and “QTL‐blood” columns is not indicative of the causality of a gene because it relies on the existence of eQTL data, not the information provided by the data. Although the “QTL‐correl” column gives more insight into the eQTL data, the default Pearson correlation coefficient cutoff significance values of −0.3 and 0.3 are quite broad, so we have included an option to allow users to modify this cutoff. In addition, eQTL data are not available for all genes of interest. For these reasons, the correlation between eQTL and GWAS data does not guarantee causality for a gene, but is still good evidence for prioritization. Gene expression data were included to account for possible increased expression of genes associated with GWAS variants in relevant cell types and tissues. GTEx transcript per million (TPM) v8 data was used to measure gene expression. We focused on brain tissue, SN tissue, and SN dopaminergic neuron data because of their established role in PD and enrichment in PD GWAS loci.^3^ However, genes do not necessarily need to be expressed in the substantia nigra or other brain tissues and cell types to increase the risk of PD.

Constraint data were included to identify genes that are intolerant to specific types of variation, suggesting conservation. Therefore, we suspect genes with significant intolerance may be causal because normal variation in these genes is unlikely. We used a significance cutoff of 0.35 for the upper limit of the 90% confidence interval of the observed/expected values, as suggested by gnomAD.^29^ It is possible that variations within genes may not be associated with PD, suggesting that low intolerance/constraint scores are not necessarily causal. Previously published burden test results were included to account for genes thought to have a significant burden of rare variants in PD.^3^ However, this does not guarantee causality because causal genes may be tagged by common variants instead. The literature count was included to quickly measure the significance of genes in previously published research. However, existing studies can easily be biased, and our automated search of the literature does not account for this. In addition, some genes are difficult to identify in automated literature searches because of nomenclature changing over time or their similarity to common names. Examples of these name complications include *SHE* and *MAL* (sometimes used for “mean axonal length” instead of the gene encoding “myelin and lymphocyte protein”).

Overall, for approximately half of all PD GWAS loci, an educated guess can be made based on the data included in our browser to choose the most likely candidate gene underlying the GWAS risk signal. Some loci have no obvious candidate (eg, loci 34, 50, and 74), but our browser may still help to prioritize candidate genes with the help of additional specific reference data. It should also be noted that there is not a “one‐fits‐all” efficient scoring system for the prioritization of genes under GWAS peaks. In addition, each included data set has clear limitations as discussed above. Therefore, we included a weight option for each data column in the evidence table. This will allow users to assign weights (0–4, with 0 for no points and 4 for 4 points) to different columns to alter the significance of the data in the final conclusion summation based on the data sets they deem most important.

In summary, we have presented an online platform that allows for prioritization of genes within PD GWAS loci. We have highlighted 2 examples (*P2RY12* and *DYRK1A*), but many other interesting gene candidates can be identified using this application. The platform is designed to be versatile, flexible, and easily expandable when more loci or data sets of interest become available. By using this platform, GWAS follow‐up studies can systematically prioritize genes based on publicly available data sets that may help to improve the design of functional experiments. In turn, this workflow could help to nominate these genes as potential therapeutic targets worthy of translating to the clinic.

## Author contributions

Design and concept: F.P.G., J.J.K., M.B.M., C.B., A.S.

App development: F.P.G., J.J.K., M.B.M.

Additional data contribution and analysis: all.

Manuscript drafting: F.P.G., X.R., C.B.

Manuscript revision: all.

## Supporting information


**Appendix** **S1**: Supporting information
